# Accurate near surface air temperature measurements are necessary to gauge large‐scale ecological responses to global climate change

**DOI:** 10.1002/ece3.3972

**Published:** 2018-05-07

**Authors:** Adam Terando, Elsa Youngsteadt, Emily Meineke, Sara Prado

**Affiliations:** ^1^ Department of Interior Southeast Climate Science Center US Geological Survey Raleigh NC USA; ^2^ Department of Entomology and Plant Pathology North Carolina State University Raleigh NC USA; ^3^ Department of Organismic and Evolutionary Biology Harvard University Cambridge MA USA; ^4^ North Carolina Cooperative Fish and Wildlife Research Unit North Carolina State University Raleigh NC USA

## Abstract

**Linked Article**: https://doi.org/10.1002/ece3.3965

Ashcroft (2018) argues that air temperature is rarely ecologically relevant at the organismal scale. We agree that air temperature is often not the best metric if the goal of a study is to characterize detailed thermobiology of specific organisms or microhabitats. The surface temperatures of a particular stratum hinge on the material's heat capacity and energy absorption potential, often resulting in significant departures from near‐surface air temperature. These departures can affect physiological processes, particularly for ectotherms, such as insects and reptiles.

However, air temperature often does correlate with organismal‐scale processes, such as phenological timing and species distributions (Thackeray et al., 2016). In addition, near‐surface air temperature, typically measured at the standard height of 2 m, is the variable for which extensive temporal and geographic observations are available and is one of the standard output variables produced by globally coordinated climate model experiments (Taylor, Stouffer, & Meehl, 2012). Therefore, even when air temperature is only an indirect predictor of a biological process, it still provides a key link between that process and climate data that are available over broad spatial and temporal scales. Such a link is much harder to make with measures such as body temperature of a given species, for which mapped historical records and future projections do not exist as they do for air temperature.

Ashcroft argues that because many species are directly exposed to radiation, using shielded sensors could result in recording temperatures that are biased cold. However, our study clearly shows (table 2, Terando, Youngsteadt, Meineke, & Prado, 2017) that temperatures recorded by radiation‐exposed, inexpensive sensors vary with sensor type, with biases ranging from 1.1°C for the HOBO Pro sensor to 2.2°C for the iButton to 3.4°C for the HOBO pendant. This indicates that the materials surrounding the sensor—stainless steel for the iButton, composite plastic for the HOBO Pendant—are susceptible to additional direct heating that affects the recorded temperature. As such, ecologists should not assume that the temperature recorded by a radiation‐exposed inexpensive sensor will correlate with the temperature experienced by an organism. And, caution is still required when selecting unshielded instruments to ensure that they have similar thermal properties to the organisms or habitat elements under study.

Finally, Ashcroft notes that the differences between temperatures at ground level and 2 m are greater than the differences between shielded and unshielded sensors, such that, from the perspective of ground‐level organisms, sensor height is a more important source of bias than shielding. We agree that sensor height is an important consideration, but it does not negate the importance of biases in air temperature introduced through inadequate shielding. We showed that even within a study, when sensors are deployed at a consistent height, bias in poorly shielded sensors varied with sun exposure and ground cover. The resulting 3–5°C differences in air temperature are the equivalent of several decades of climate change, or the difference between starkly contrasting socioeconomic scenarios of greenhouse gas management. Such biases within an ecological study could be misleading if the temperatures were accepted as accurate and interpreted relative to climate projections.

We appreciate the points raised by Ashcroft and believe this is an important conversation. We also in no way want to argue against using thermal variables other than air temperature, when these are the most salient to research questions. As Ashcroft noted, about one‐third of the studies we examined monitored air temperature with inexpensive data loggers, and it was this one‐third that we evaluated for shielding practices. We do not suggest that the other two‐thirds should have measured air temperature instead of soil, surface, or body temperature. Rather, we suggest that when ecologists do measure air temperature, we must take more care to do so accurately. As we move deeper into the Anthropocene, and as we observe, model, and predict effects of climate change on organisms, accurate air temperature data will be critical. This will improve assessments of ecological costs of anthropogenic greenhouse gas emissions and aid in the development of adaptation strategies. Innovation and creativity are the lifeblood of scientific advancement and should be encouraged at every opportunity. However, with the stakes so high, we also must be vigilant in holding ourselves to a high standard of meticulousness and rigor when documenting the myriad ways that human‐caused warming is affecting the ecology of the planet.

## CONFLICTS OF INTEREST

None declared.
